# Global, regional, and national burden of oral cancer from 1990 to 2021: Analysis of risk factors and prediction of trends in 2050

**DOI:** 10.1097/MD.0000000000050197

**Published:** 2026-09-11

**Authors:** Jiangbo Xin, Weiling Chen, Dongzhen Li, Haoxuan Sun

**Affiliations:** a Department of Oral and Maxillofacial Surgery, Hebei Eye Hospital, Xingtai, P.R. China.

**Keywords:** autoregressive integrated moving average model, Global Burden of Disease, oral cancer, risk factors, Socio-demographic Index

## Abstract

The Global Burden of Disease (GBD) project provides valuable insights into the global, regional, and national burdens of oral cancer (OC). This study aimed to assess the burden of OC from 1990 to 2021, analyze associated risk factors, and project future trends up to 2050. Data on OC were derived from the GBD 2021 report using the International Classification of Diseases, Tenth Revision codes C00–C08. Incidence, mortality, and disability-adjusted life years (DALYs) were estimated using DisMod-MR 2.1. Age-standardized rates were applied to assess temporal patterns. An autoregressive integrated moving average model was used to forecast future trends, with model selection and performance evaluated using standard diagnostic criteria. Potential risk factors, including smoking, alcohol consumption, and tobacco use, were further examined using Mendelian randomization analyses. From 1990 to 2021, global incidence, mortality, and DALYs attributable to OC increased, particularly in regions with lower Socio-demographic Index (SDI) levels, while high-SDI regions showed relatively stable or declining mortality. Smoking remained a major contributor to the OC burden, with alcohol use and other behavioral risks also playing important roles. Autoregressive integrated moving average projections suggested a continued increase in DALYs and deaths by 2050; however, these forecasts depend on current exposure and reporting assumptions. OC continues to impose a substantial global health burden, especially in low- and middle-income countries. Although projections indicate a likely increase in the future disease burden, these estimates should be interpreted with caution due to uncertainties in GBD data and long-term modeling. Strengthened prevention and tobacco control strategies remain essential to mitigating future risk.

## 1. Introduction

Oral cancer (OC) is the predominant carcinoma of the head and neck.^[1,2]^ Despite OC being a serious global public health issue, statistical data on incidence, mortality, and disability-adjusted life years (DALYs) reveal significant geographical differences worldwide.^[3–6]^ It is ranked 15th in cancer incidence in the United States and 10th in Europe. Asian developing countries exhibit a high prevalence of OC.^[7–12]^ Although OC is not among the predominant cancers globally and has seen a decline in many developed countries in recent years, its mortality rate in developing countries remains significant.^[13,14]^ Numerous differences arise from variations in population habits, healthcare service availability, and preventive education across various countries. Therefore, gathering such data is advantageous for examining the effects of established pathogenic factors and identifying potential pathogenic factors. Comparing OC indicators and their long-term trends among different countries or regions is highly beneficial for guiding the development of various health policies and screening initiatives aimed at the prevention and management of OC. Moreover, reliable and precise reporting on disease epidemiological patterns and long-term trends across various locations can provide policymakers with the necessary information to deploy resources judiciously. The ongoing variations in the incidence and mortality rates of OC require further clarification. Previous studies have documented the global incidence and mortality rates of OC, however, detailed information regarding its spatial distribution and long-term trends remains unreported.

The Global Burden of Disease (GBD) study provides standardized methodological frameworks and harmonized datasets that enable robust comparisons of OC burden across countries, regions, and socio-demographic contexts. Previous studies have used GBD or national cancer registry data to describe trends in incidence and mortality; however, many analyses have focused on limited regions, single indicators, or shorter time frames, and often lacked integration of risk factor attribution or causal inference approaches. Moreover, few studies have combined historical burden assessment with projections of future disease trends, which are essential for planning resource allocation and evaluating long-term prevention strategies.

Using GBD 2021 data, we assess global, regional, and national trends in incidence, mortality, and DALYs of OC from 1990 to 2021, evaluate disparities across Socio-demographic Index (SDI) levels, quantify the contributions of key behavioral risk factors, and apply forecasting models to estimate future burden up to 2050. By combining temporal trend analysis, risk factor attribution, and long-term projection, this study aims to provide comprehensive evidence to support OC prevention, tobacco control, and health policy planning at national and global levels.

## 2. Materials and methods

### 2.1. Overview and global data sources

In GBD 2021, the *International Statistical Classification of Diseases and Health-related Questions, 10th Revision* was employed for classification and coding, with the disease classification codes for OC designated as C00–C08. This manuscript adhered to the *Guidelines for Accurate and Transparent Health Estimate Reporting* statement. The 2021 GBD cycle incorporates data up to the 2021 data, covering 204 countries and territories across 21 regions.

We acquired incidence, prevalence, mortality, and DALY estimates for OC from the GBD database. DisMod-MR 2.1, a Bayesian meta-regression tool, was employed to generate estimates based on age, gender, year, and nation/region. DALY is defined as the sum of years of life lost and years lived with disability (YLD). Years of life lost were computed as the product of the estimated number of death and the standardized life expectancy corresponding to age at death. YLD was calculated by multiplying the prevalence of the individual consequences of the disease by its corresponding disability weight. GBD provides uncertainty estimates calculated from 1000 posterior draws of the DisMod-MR models. We report 95% uncertainty intervals for incidence, mortality, and DALYs, defined as the 2.5th and 97.5th percentiles of these posterior distributions.

The SDI serves as a holistic metric representing the developmental state of a country. It considers factors such as per capita income, average years of schooling, and the fertility rate among young women. The SDI value range spans from 0 to 1, categorized as follows: high (0.805129–1), high-middle (0.689504–0.805129), intermediate (0.607679–0.689504), low-middle (0.454743–0.607679), and low (0–0.454743).

This study used de-identified, publicly available, aggregated population estimates from the GBD 2021 database, with no access to individual-level data. According to international guidance on secondary analyses of anonymized data, institutional review board approval and patient consent were not required. All primary data sources included in the GBD framework obtained the necessary ethical approvals before contributing data.

### 2.2. Attributable risk factors

The GBD 2021 study provides a comprehensive assessment of the attributable burden of disease associated with 88 risk factors and their combinations across global, regional, and national levels. Attributable counts and ASRM, DALY, and YLD were estimated for 3 primary categories of risk factors: all risk factors, tobacco use, and alcohol consumption. According to the comparative risk assessment method, the population attributable fraction (PAF) was calculated using the attributable count of specific risk factors. The PAF signifies the expected reduction in the existing disease burden that could result from altering the population’s exposure to risk factors to an alternative or hypothetical risk distribution scenario. The quantified burden of each factor was determined by multiplying the respective measurement by PAF. In GBD 2021, the PAF estimation is based on a theoretical minimum risk exposure level, which reflects the exposure distribution associated with the lowest population risk rather than a zero-exposure assumption. For closely related or overlapping exposures, such as smoking and overall tobacco use, GBD applies a hierarchical framework to avoid double counting and ensures that attributable fractions do not exceed the total burden. Uncertainty in risk–outcome associations, exposure estimation, and model specification is propagated using 1000 posterior draws, and the resulting attributable estimates are reported with 95% uncertainty intervals.

### 2.3. Mendelian randomization (MR) study design

The effect of various factors on the risk of lip and OC was investigated using a two-sample MR approach. The study design was based on the following assumptions: the genetic variants are strongly associated with the exposure; the genetic variants are independent of any potential confounders, i.e. the genetic variants only work through the exposure and are unaffected by the confounders; the genetic variants affect the outcome only through their effect on the exposure and not directly.

### 2.4. Sources of Mendelian randomized OC data

The OC data from genome-wide association studies (GWAS) are from https://gwas.mrcieu.ac.uk/, the dataset is ieu-b-4962, the number of samples is 372,855 (including 839 OC patients and 372,016 controls), the number of SNPs is 9185,233, and the ethnicity is European. Exposure factor data: smoking ieu-b-2134, Tobacco ukb-b-5115; Alcohol use ukb-b-391 (because no relevant datasets were found for Behavioral risks and Chewing tobacco, no MR risk factor analysis was performed). All MR analyses were conducted using publicly available summary-level GWAS summary statistics.

### 2.5. Instrument variables

Based on GWAS analysis, SNPs associated with the variable factors were selected as instrument variables. For each independent SNP, i.e. a SNP that is not in linkage disequilibrium, we set a genome-wide significance threshold (*P* < 5 × 10^−8^, *r*2 < 0.001, kb = 10,000). When a variant was not included in the results GWAS, we replaced it with the best possible substitute value from the 1000G-EUR data (*r*2 > 0.8). When no replacement value is available, we exclude the variant from the analysis. Palindromic SNPs are deleted, and SNPs with a minor allele frequency < 0.01 are also excluded. Use the F-statistic assessment tool to evaluate the strength of the instrument variable. Retain instrument variables with an F > 10, F = se2/beta2, and SNPs with F > 10 were retained to minimize weak instrument bias.

### 2.6. Statistical analysis

Excel 2019 was utilized to compile and analyze the number of deaths, ASRM, DALY, and age-standardized DALY rate of OC across various years, age groups, and genders in China from 1990 to 2019. The χ^2^ test was employed to analyze gender differences in attributable number of deaths and DALYs, as well as their rates of change. The test level was bilateral α = 0.05. The Joinpoint (version 4.7.0) of the National Cancer Institute of the United States was used with the default setting that includes the log-linear regression model to calculate the average APC and 95% confidence interval for the global mortality and age-standardized DALY rates from 1990 to 2021, with the trend of changing analyzed. The one-sample t-test was employed for the trend analysis. The significance level for the test was bilateral α = 0.05. The Bayesian age-period-cohort model in R software (version 4.2.2) was employed to forecast global OC mortality, DALYs, and YLDs from 2020 to 2034. Joinpoint regression was applied to quantify temporal trends and to identify potential turning points in incidence and mortality trajectories, whereas the Bayesian age–period–cohort model was used to disentangle age, period, and cohort effects and to provide short-term projections.

China-specific analyses were conducted to provide a detailed country-level example for a high-burden setting, while global and SDI-stratified analyses were performed to support international comparison and broader policy relevance.

Inverse variance weighted, MR Egger, weighted median, simple mode, and weighted mode were utilized to perform two-sample and multivariate MR (MVMR) analyses for OC and GBD risk factors, including tobacco use, alcohol consumption, and smoking. To assess the reliability of the analysis results, a sensitivity analysis was conducted on the MR results, and IVW and MR-Egger regression methods were used to calculate the Cochran *Q* statistic. The results were assessed for heterogeneity, or the lack of heterogeneity (*P* > .05) indicated that the results were dependable. The function MR_pleiotropy_test from the R package TwoSampleMR was utilized to assess the presence of horizontal pleiotropy. A *P* value exceeding .05 signifies dependable results. Leave-one-out analysis assessed the influence of the remaining instrumental variables on the outcome by sequentially removing each instrumental variable. If the result significantly alters upon the exclusion of a specific variable, it suggests that the outcome was sensitive to this factor. All the above analyses were performed by using R software (version 4.2.2).

## 3. Results

### 3.1. Relative changes in the burden of OC

**Figure 1.**
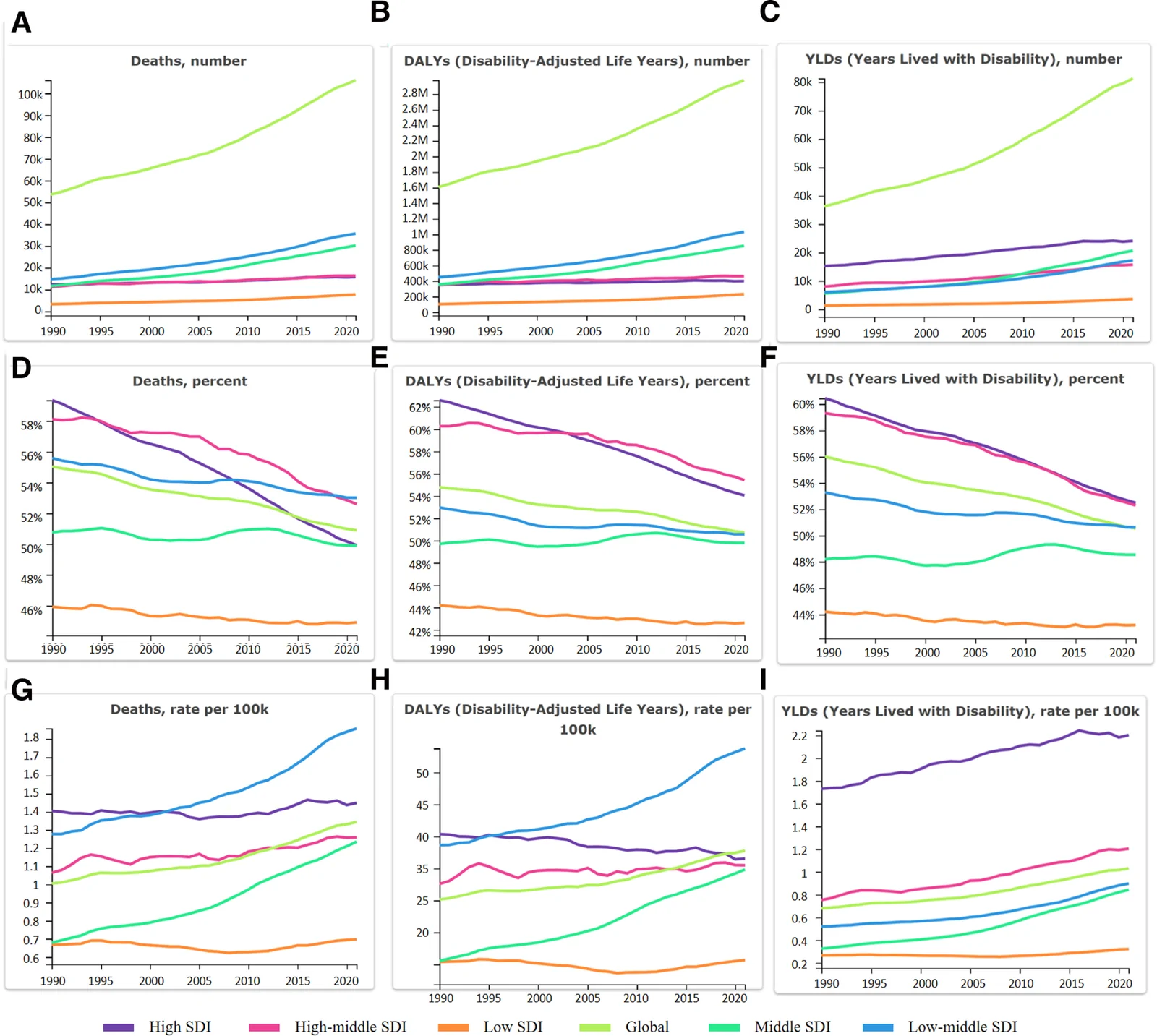
Trends in oral cancer burden by SDI level, 1990–2021 GBD datasets, this figure shows observational outputs from the GBD 2021 study only. (A–C) Deaths, DALYs, and YLDs number; (D–F) Deaths, DALYs, and YLDs percent, population attributable fraction attributable to all risk factors; and (G–I) Deaths, DALYs, and YLDs rate (per 100,000 population). DALYs = disability-adjusted life years, GBD = Global Burden of Disease, SDI = Sociodemographic Index, YLDs = years lived with disability.

In the GBD database, a search for OC revealed that from 1990 to 2021, the total number of death of OC patients and the number of death per 100,000 individuals across various SDI regions globally exhibited an upward trend, whereas the overall mortality rate demonstrated a declining trend, as illustrated in Figure 1 and Table 1. The results are presented as age-standardized rates, because age-standardized rates better reflect true epidemiological changes independent of population aging and growth.

**Table 1:** Relative changes in the burden of oral cancer.

Risk	Deaths rate% (95% UI)	DALYs (95% UI)	YLDs (95% UI)
Global			
1990	1.00505324277999 (1.13817211381–0.87881884291)	30.15730247923 (34.00186138257–26.48094776707)	0.67927826356 (0.8883586193–0.50030439203)
2021	1.34425719629 (1.54558185619999–1.16503748853999)	37.77415418061 (43.37373519994–32.56845684754)	1.03061800929 (1.38894243899–0.75261991685)
Low SDI			
1990	0.66707332774 (0.80953428889–0.53629860618)	20.40197244438 (24.87840380753–16.33070941848)	0.26546862317 (0.3688255155–0.18885793585)
2021	0.69696585961 (0.837796713929999–0.558558026149999)	20.68653874172 (24.94542678641–16.40544641685)	0.32163252171 (0.45405475922–0.22175495191)
Low-middle SDI			
1990	1.276984278 (1.50488373262–1.06900673152)	38.63249883685 (45.69981345603–32.22283201543)	0.51912464655 (0.70561502444–0.37848268123)
2021	1.858506186 (2.16621765914–1.57409884262)	53.71695883319 (62.50929756731–44.85209185823)	0.89720819034 (1.2180135519–0.63925966935)
Middle SDI			
1990	0.67908690247 (0.77701537793–0.58849276073)	20.55547838621 (23.46168418091–17.79180980075)	0.3257024678 (0.43835719039–0.23537497251)
2021	1.23506581422 (1.46392486422–1.04550661617)	34.83322504315 (41.5394777923–29.28383863605)	0.8430389233 (1.15117429137999–0.59976338592)
High-middle SDI			
1990	1.064117105 (1.19414423623999–0.925181159609999)	32.57978704 (36.28537246835–28.58452401602)	0.7521266515 (0.99404795094–0.55448911025)
2021	1.258742513 (1.48754580852–1.05185986236)	35.49208577843 (41.62659447656–29.92878396626)	1.20406668139 (1.62932755988999–0.86631237821)
High SDI			
1990	1.40420430838 (1.60073438722–1.19369590776)	40.35895760284 (45.61501071358–34.713378838)	1.73050107074 (2.29212570173–1.23839150438)
2021	1.44832072602 (1.68691458975–1.20272750225)	36.52639269948 (41.8670791885–30.70365885637)	2.20168864632 (2.9585581011–1.57008365656999)

DALYs = disability-adjusted life years, SDI = Socio-demographic Index, UI = uncertainty intervals, YLDs = years lived with disability.

### 3.2. Risk factor analysis

**Figure 2.**
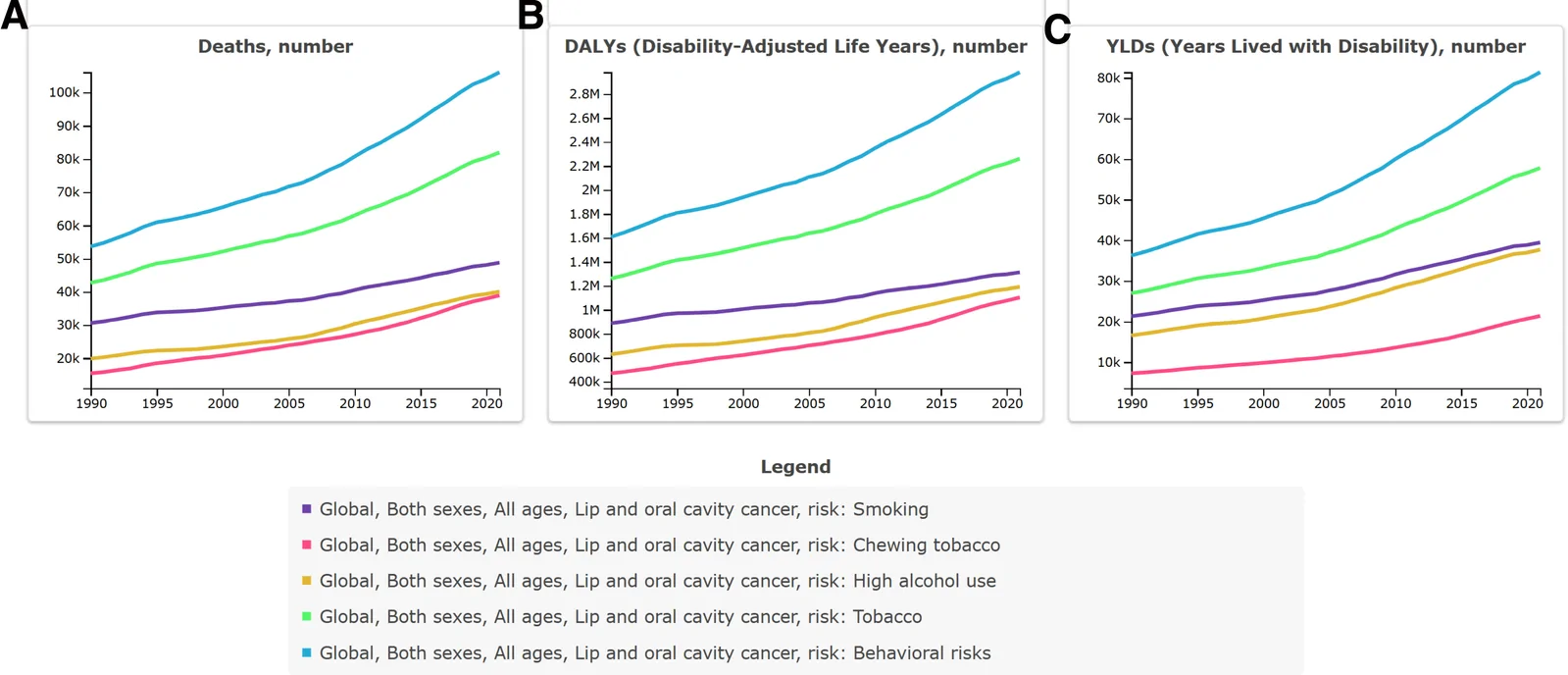
The impact of risk factors on (A) death, (B) DALYs, and (C) YLDs of oral cancer. DALYs = disability-adjusted life years, YLDs = years lived with disability.

Subsequently, the main risk factors influencing OC were identified. Table 2 indicates that the risk factors affecting OC encompass tobacco use, behavioral risk, alcohol consumption, chewing tobacco, and smoking. Among them, behavioral risk was the main cause of death, DALY, and YLD associated with OC. The death, DALY, and YLD of OC related to behavioral risks all showed an upward trend from 1990 to 2021 (Fig. 2). Since 1990, the DALY attributed to OC due to behavioral risk rose from 30.15730248 to 37.77415418. The influence of tobacco and smoking on death, DALY, and YLD associated with OC was surpassed only by behavioral risk.

**Table 2:** Major risk factors for oral cancer.

Risk	Deaths rate% (95% CI)	DALYs (95% CI)	YLDs (95% CI)
Tobacco			
1990	0.800675099 (0.94803168039–0.65064399468)	23.64135678 (0.94803168039–0.65064399468)	0.504929681 (0.69992196072–0.3531460495)
2021	1.038436427 (1.24291893939–0.83559737769)	28.64318276 (34.24479065506–23.02490253991)	0.73224459 (1.03729537137–0.51578392206)
Behavioral risks			
1990	1.005053243 (1.13817211381–0.87881884291)	30.15730248 (34.00186138257–26.48094776707)	0.679278264 (0.8883586193–0.50030439203)
2021	1.344257196 (1.54558185619999–1.16503748853999)	37.77415418 (43.37373519994–32.56845684754)	1.030618009 (1.38894243899–0.75261991685)
Alcohol use			
1990	0.372420838 (0.45131738595–0.2919558797)	11.78343647 (14.15351667126–9.25303606055)	0.310243261 (0.42209611614–0.2114456073)
2021	0.507367113 (0.62880335244–0.389822228369999)	15.09353894 (18.67876009515–11.48382493314)	0.477368422 (0.64932851198–0.326339686629999)
Chewing tobacco			
1990	0.289446751 (0.36836255645–0.2166212331)	8.801419496 (11.22916438008–6.54646807566)	0.13514438226 (0.19156062887–0.08972581175)
2021	0.49299083 (0.94803168039–0.37704801542)	13.97502235 (17.28481073946–10.51070955233)	0.270154531 (0.39164296431–0.17704176516)
Smoking			
1990	0.573380866 (0.60715929754–0.41195312823)	16.63904121 (21.0072054317799–12.02639623543)	0.398770428 (0.5714534535–0.25866108005)
2021	0.618052158 (0.802993577659–0.426065811429)	16.63227242 (21.50513465946–11.5396950739599)	0.499780761 (0.73336472984–0.31962062784)

CI = confidence interval, DALYs = disability-adjusted life years, YLDs = years lived with disability.

### 3.3. MVMR analysis

The analysis of previous GBD risk factors indicated that behavioral risks, tobacco use, smoking, and alcohol consumption all played a gradually increasing role in the burden of OC. To have a deeper comprehension of the risk factors that affected OC Table 3 presents the MVMR analysis subsequent to the adjustment for major variables impacting OC, including tobacco use, alcohol consumption, and smoking. Results indicated that genetically proxied smoking was associated with OC risk (OR = 2.15, 95% confidence interval = 1.54–3.88, *P* = 5.14E−04). All sensitivity analyses are presented in Figure S1, Supplemental Digital Content 1. These MR findings strengthen the evidence supporting an association between smoking and OC risk; however, they should still be interpreted in light of underlying MR assumptions and potential residual pleiotropy.

**Table 3:** Results of MVMR analysis.

Exposure	Outcome	Method	nsnp	beta	se	pval	OR (95% CI)
Smoke	Oral cancer	MR Egger	87	0.74	0.002	.0009	2.03 (1.68 to 2.39)
Smoke	Oral cancer	Weighted median	87	0.41	0.001	.0020	1.14 (1.01 to 1.53)
Smoke	Oral cancer	IVW	87	0.78	0.0006	.0011	2.15 (1.54 to 3.88)
Smoke	Oral cancer	Simple mode	87	0.38	0.002	.0032	1.04 (1.03 to 2.46)
Smoke	Oral cancer	Weighted mode	87	0.38	0.002	.0024	1.05 (1.00 to 2.55)
Tobacco	Oral cancer	MR Egger	3	-0.05	0.14	.7846	−0.14 (−0.00 to 6.30)
Tobacco	Oral cancer	Weighted median	3	0.04	0.03	.1790	0.14 (0.00 to 6.46)
Tobacco	Oral cancer	IVW	3	0.03	0.03	.2319	0.10 (0.00 to 2.43)
Tobacco	Oral cancer	Simple mode	3	0.05	0.04	.3716	0.12 (0.00 to 4.92)
Tobacco	Oral cancer	Weighted mode	3	0.05	0.04	.3634	0.14 (0.01 to 7.85)
Alcohol	Oral cancer	MR Egger	6	1.30	0.86	.2070	3.53 (0.94 to 7.94)
Alcohol	Oral cancer	Weighted median	6	0.008	0.06	.8986	0.24 (0.00 to 2.09)
Alcohol	Oral cancer	IVW	6	0.004	0.09	.9636	0.11 (0.00 to 1.73)
Alcohol	Oral cancer	Simple mode	6	0.06	0.147	.6945	0.17 (0.00 to 1.18)
Alcohol	Oral cancer	Weighted mode	6	0.02	0.12	.8534	0.07 (0.00 to 1.05)

CI = confidence interval, MR = Mendelian randomization, MVMR = multivariate MR.

### 3.4. Impact of risk factors on the burden of OC

**Figure 3.**
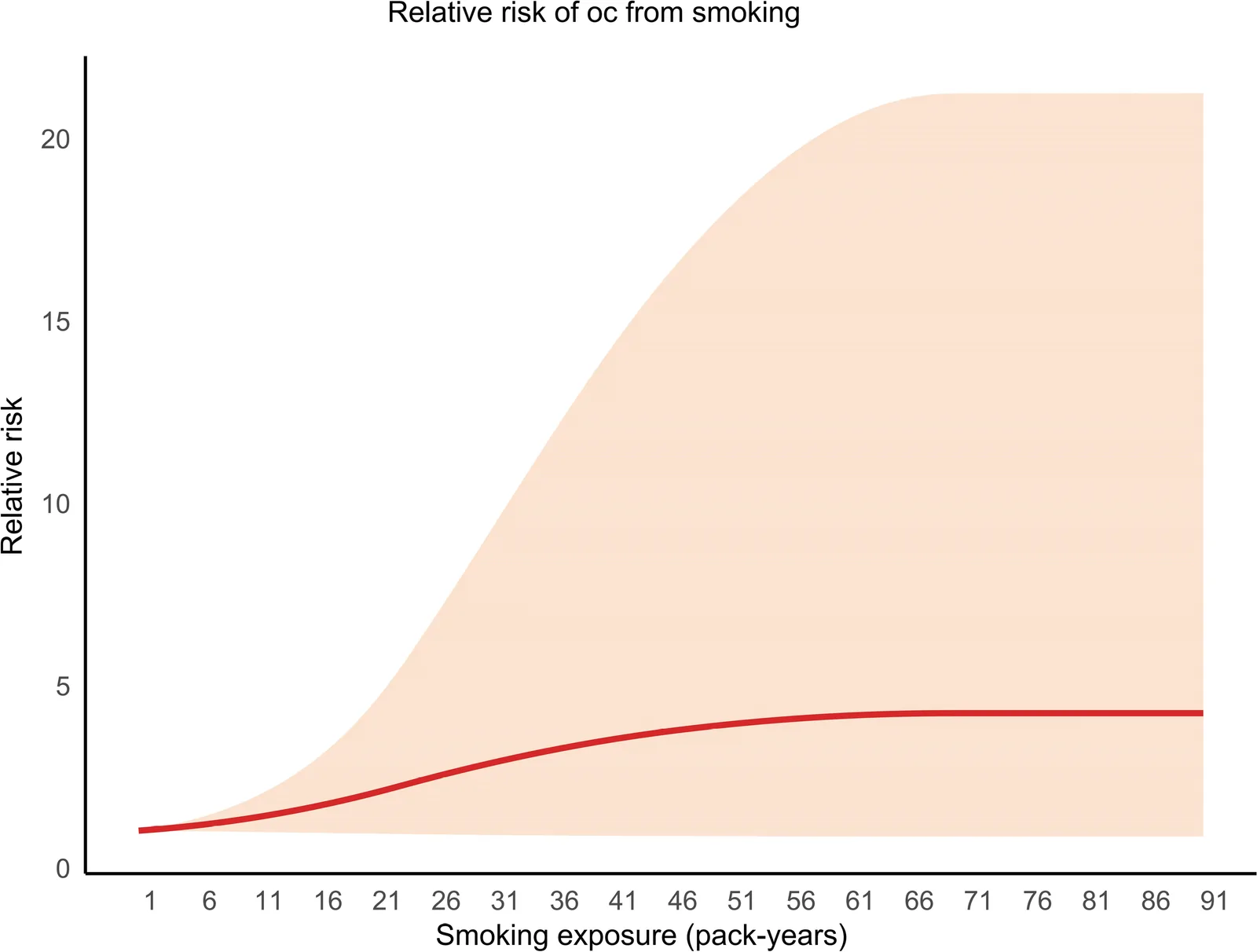
The impact of smoking on oral cancer. OC = oral cancer.

MR analysis revealed a causal relationship between smoking and OC (*P* < .05). No causal relationship was identified between alcohol consumption and tobacco use. Therefore, in the prediction of GBD, we selected smoking as a risk factor and forecasted the disease burden of OC in 2050. We searched for the global risk of smoking on OC in the GBD database. Table 4 illustrates the correlation between the risk of OC and the average yearly quantity of smoking. As seen in Figure 3.

**Table 4:** The effect of smoking on oral cancer.

Risk name	Outcome name	Log relative risk of outcome (lower fixed & random effects uncertainty)	Log relative risk of outcome (lower fixed effects uncertainty)	Log relative risk of outcome	Log relative risk of outcome (upper fixed effects uncertainty)	Log relative risk of outcome (upper fixed & random effects uncertainty)	Risk
Smoking	OC	0	0	0	0	0	0
Smoking	OC	−0.002615332	0.015365931	0.021613126	0.027860322	0.045841585	0.909090909
Smoking	OC	−0.00544492	0.031990689	0.04499687	0.058003052	0.095438661	1.818181818
Smoking	OC	−0.008474448	0.049790158	0.070032917	0.090275676	0.148540282	2.727272727
Smoking	OC	−0.011689355	0.068678787	0.096600935	0.124523083	0.204891226	3.636363636
Smoking	OC	−0.015075019	0.088570673	0.124580095	0.160589517	0.264235209	4.545454545
Smoking	OC	−0.018616924	0.109380524	0.153850429	0.198320334	0.326317781	5.454545455
Smoking	OC	−0.022300801	0.131024508	0.184294024	0.237563539	0.390888848	6.363636364
Smoking	OC	−0.026112754	0.153420978	0.215796034	0.278171091	0.457704822	7.272727273
Smoking	OC	−0.030039357	0.176491057	0.248245519	0.319999981	0.526530395	8.181818182
Smoking	OC	−0.034067739	0.20015912	0.281536104	0.362913087	0.597139946	9.090909091
Smoking	OC	−0.038185641	0.224353144	0.315566485	0.406779826	0.669318611	10
Smoking	OC	−0.042381462	0.249004965	0.350240787	0.451476608	0.742863035	10.90909091
Smoking	OC	−0.046644284	0.27405044	0.385468787	0.496887133	0.817581857	11.81818182
Smoking	OC	−0.050963887	0.299429525	0.421166029	0.542902532	0.893295944	12.72727273
Smoking	OC	−0.055330752	0.325086285	0.457253838	0.589421392	0.969838429	13.63636364
Smoking	OC	−0.05973605	0.350968852	0.493659259	0.636349667	1.047054569	14.54545455
Smoking	OC	−0.064171629	0.37702933	0.530314924	0.683600517	1.124801476	15.45454545
Smoking	OC	−0.068629991	0.403223669	0.567158871	0.731094074	1.202947734	16.36363636
Smoking	OC	−0.073104267	0.429511508	0.604134332	0.778757157	1.281372932	17.27272727
Smoking	OC	−0.077588186	0.455856	0.64118948	0.82652296	1.359967147	18.18181818
Smoking	OC	−0.082074457	0.482214306	0.678264057	0.874313809	1.438602571	19.09090909
Smoking	OC	−0.086547703	0.508496091	0.715231002	0.921965914	1.517009707	20
Smoking	OC	−0.091000201	0.534655975	0.752026487	0.969396999	1.595053174	20.90909091
Smoking	OC	−0.095410407	0.560567383	0.788472476	1.016377568	1.672355359	21.81818182
Smoking	OC	−0.099714629	0.585856097	0.824042606	1.062229115	1.747799841	22.72727273
Smoking	OC	−0.103845435	0.610125935	0.858179624	1.106233313	1.820204683	23.63636364
Smoking	OC	−0.107772039	0.633196016	0.890629112	1.148062207	1.889030262	24.54545455
Smoking	OC	−0.111501295	0.655106615	0.921447716	1.187788816	1.954396726	25.45454545
Smoking	OC	−0.115043262	0.675916833	0.950718565	1.225520298	2.016480392	26.36363636
Smoking	OC	−0.118409617	0.695695273	0.978538158	1.261381042	2.075485933	27.27272727
Smoking	OC	−0.121611084	0.714504935	1.004995102	1.29548527	2.131601288	28.18181818
Smoking	OC	−0.12465732	0.732402573	1.030169231	1.327935889	2.184995782	29.09090909
Smoking	OC	−0.127557055	0.749439465	1.054132666	1.358825867	2.235822388	30
Smoking	OC	−0.130318195	0.765662063	1.076950747	1.388239431	2.284219688	30.90909091
Smoking	OC	−0.13294792	0.781112559	1.098682819	1.416253079	2.330313557	31.81818182
Smoking	OC	−0.135452768	0.795829366	1.119382912	1.442936459	2.374218593	32.72727273
Smoking	OC	−0.137838706	0.809847532	1.139100325	1.468353118	2.416039356	33.63636364
Smoking	OC	−0.140111187	0.823199097	1.157880122	1.492561147	2.455871431	34.54545455
Smoking	OC	−0.142275203	0.835913404	1.175763576	1.515613748	2.493802355	35.45454545
Smoking	OC	−0.144335337	0.848017367	1.192788543	1.53755972	2.529912424	36.36363636
Smoking	OC	−0.146295797	0.859535708	1.208989799	1.558443889	2.564275394	37.27272727
Smoking	OC	−0.148160452	0.870491167	1.224399324	1.578307482	2.5969591	38.18181818
Smoking	OC	−0.149932865	0.880904677	1.239046566	1.597188455	2.628025997	39.09090909
Smoking	OC	−0.151616321	0.89079553	1.252958659	1.615121787	2.657533638	40
Smoking	OC	−0.153213847	0.900181516	1.266160625	1.632139733	2.685535096	40.90909091
Smoking	OC	−0.154728236	0.909079048	1.278675551	1.648272054	2.712079338	41.81818182
Smoking	OC	−0.156162378	0.917505101	1.290527312	1.663549523	2.737217001	42.72727273
Smoking	OC	−0.157520136	0.925482374	1.301747837	1.678013299	2.761015809	43.63636364
Smoking	OC	−0.158805361	0.933033491	1.312368946	1.691704402	2.783543253	44.54545455
Smoking	OC	−0.160022045	0.940181908	1.322423634	1.704665361	2.804869314	45.45454545
Smoking	OC	−0.161175483	0.946958738	1.331955662	1.716952587	2.825086808	46.36363636
Smoking	OC	−0.162270727	0.953393653	1.341006766	1.72861988	2.84428426	47.27272727
Smoking	OC	−0.1633112	0.95950677	1.349605241	1.739703712	2.862521681	48.18181818
Smoking	OC	−0.164298739	0.965308888	1.357766276	1.750223664	2.879831291	49.09090909
Smoking	OC	−0.16523467	0.970807789	1.365500818	1.760193848	2.896236307	50
Smoking	OC	−0.166120018	0.976009497	1.372817341	1.769625185	2.9117547	50.90909091
Smoking	OC	−0.166955737	0.980919622	1.379723733	1.778527845	2.926403204	51.81818182
Smoking	OC	−0.167742716	0.98554338	1.386227333	1.786911285	2.940197381	52.72727273
Smoking	OC	−0.168481778	0.989885614	1.392334951	1.794784289	2.953151681	53.63636364
Smoking	OC	−0.169173689	0.993950813	1.398052904	1.802154995	2.965279496	54.54545455
Smoking	OC	−0.169819153	0.997743128	1.403387028	1.809030928	2.976593208	55.45454545
Smoking	OC	−0.170418823	1.001266386	1.408342707	1.815419028	2.987104237	56.36363636
Smoking	OC	−0.170973298	1.004524105	1.412924888	1.821325672	2.996823074	57.27272727
Smoking	OC	−0.171483124	1.007519503	1.417138101	1.826756698	3.005759326	58.18181818
Smoking	OC	−0.171948802	1.010255515	1.420986469	1.831717424	3.013921741	59.09090909
Smoking	OC	−0.172370784	1.012734795	1.42447373	1.836212664	3.021318243	60
Smoking	OC	−0.172749476	1.014959732	1.42760324	1.840246748	3.027955957	60.90909091
Smoking	OC	−0.173085239	1.016932454	1.430377995	1.843823535	3.033841229	61.81818182
Smoking	OC	−0.173378394	1.018654834	1.432800629	1.846946423	3.038979651	62.72727273
Smoking	OC	−0.173629217	1.020128501	1.434873431	1.849618361	3.043376079	63.63636364
Smoking	OC	−0.173837944	1.021354838	1.43659835	1.851841861	3.047034643	64.54545455
Smoking	OC	−0.174004769	1.022334994	1.437976999	1.853619004	3.049958767	65.45454545
Smoking	OC	−0.174129855	1.023069915	1.43901071	1.854951505	3.052151275	66.36363636
Smoking	OC	−0.174213459	1.023561111	1.439701608	1.855842105	3.053616675	67.27272727
Smoking	OC	−0.174258217	1.023824083	1.440071494	1.856318905	3.054401205	68.18181818
Smoking	OC	−0.174275415	1.023925125	1.440213616	1.856502107	3.054702646	69.09090909
Smoking	OC	−0.174279073	1.023946615	1.440243843	1.856541072	3.054766759	70
Smoking	OC	−0.174279558	1.023949468	1.440247856	1.856546244	3.054775269	70.90909091
Smoking	OC	−0.17427981	1.023950947	1.440249936	1.856548925	3.054779682	71.81818182
Smoking	OC	−0.174280062	1.023952426	1.440252016	1.856551607	3.054784094	72.72727273
Smoking	OC	−0.174280313	1.023953905	1.440254096	1.856554288	3.054788506	73.63636364
Smoking	OC	−0.174280565	1.023955383	1.440256176	1.856556969	3.054792918	74.54545455
Smoking	OC	−0.174280817	1.023956862	1.440258257	1.856559651	3.05479733	75.45454545
Smoking	OC	−0.174281068	1.023958341	1.440260337	1.856562332	3.054801742	76.36363636
Smoking	OC	−0.17428132	1.02395982	1.440262417	1.856565014	3.054806154	77.27272727
Smoking	OC	−0.174281572	1.023961299	1.440264497	1.856567695	3.054810566	78.18181818
Smoking	OC	−0.174281824	1.023962778	1.440266577	1.856570377	3.054814978	79.09090909
Smoking	OC	−0.174282075	1.023964257	1.440268657	1.856573058	3.05481939	80
Smoking	OC	−0.174282327	1.023965736	1.440270738	1.856575739	3.054823802	80.90909091
Smoking	OC	−0.174282579	1.023967215	1.440272818	1.856578421	3.054828214	81.81818182
Smoking	OC	−0.17428283	1.023968694	1.440274898	1.856581102	3.054832626	82.72727273
Smoking	OC	−0.174283082	1.023970172	1.440276978	1.856583784	3.054837038	83.63636364
Smoking	OC	−0.174283334	1.023971651	1.440279058	1.856586465	3.05484145	84.54545455
Smoking	OC	−0.174283586	1.02397313	1.440281138	1.856589146	3.054845862	85.45454545
Smoking	OC	−0.174283837	1.023974609	1.440283218	1.856591828	3.054850274	86.36363636
Smoking	OC	−0.174284089	1.023976088	1.440285299	1.856594509	3.054854686	87.27272727
Smoking	OC	−0.174284341	1.023977567	1.440287379	1.85659719	3.054859098	88.18181818
Smoking	OC	−0.174284592	1.023979046	1.440289459	1.856599872	3.05486351	89.09090909
Smoking	OC	−0.174284844	1.023980524	1.440291539	1.856602553	3.054867922	90

OC = oral cancer.

### 3.5. Prediction of OC in 2050

**Figure 4.**
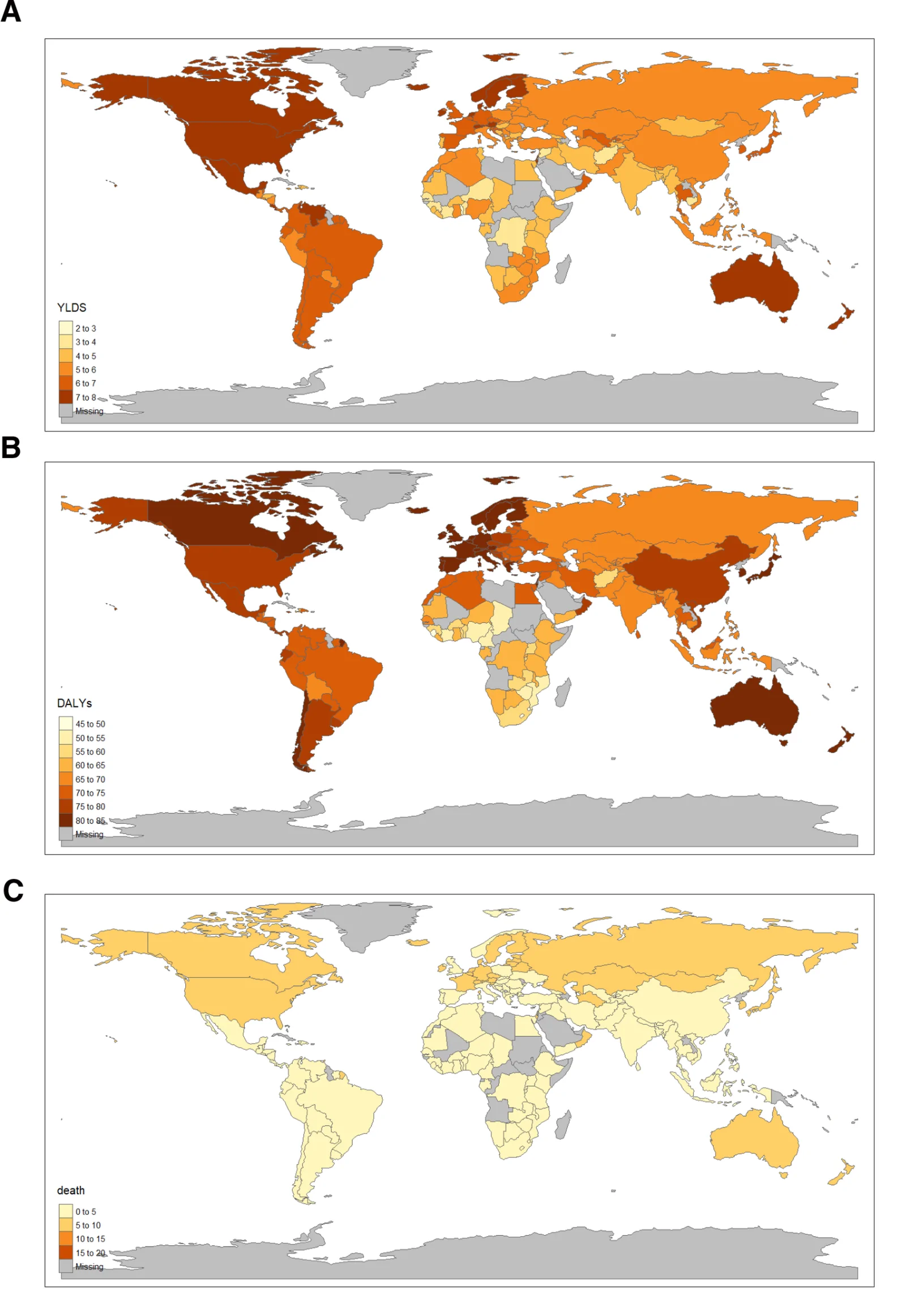
Global burden of oral cancer attributable to smoking across 204 countries/regions in 2050. (A) Mortality; (B) YLDS; and (C) DALYs. DALYs = disability-adjusted life years, YLDs = years lived with disability.

**Figure 5.**
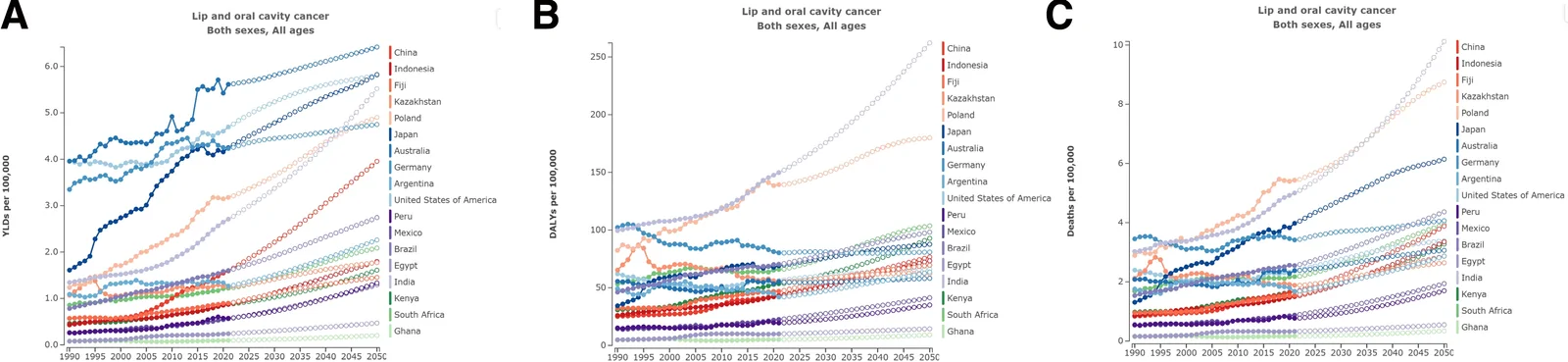
ARIMA model prediction of oral cancer burden attributable to smoking from 2020 to 2050, ARIMA model prediction of (A) YLDs, (B) DALYs, and (C) the number of deaths. ARIMA = autoregressive integrated moving average, DALYs = disability-adjusted life years, YLDs = years lived with disability.

According to the previous findings, GBD predictions indicated that smoking will result in anticipated deaths, DALYs, and YLDs from OC in 2050 across 204 countries globally. The burden of smoking on OC across 204 countries globally in 2050 is illustrated in Figure 4. The anticipated developmental trends of the top 20 countries with the most significant consequences are illustrated by curve graphs (Fig. 5). These autoregressive integrated moving average-based projections are scenario estimates and assume relatively stable future exposure patterns and reporting conditions. Therefore, they should be interpreted as indicative trends rather than precise predictions.

## 4. Discussion

OC is a disease of considerable public health importance worldwide.^[15]^ Particularly in certain low- and middle-income countries, the incidence and mortality rates persist in escalating.^[16]^ The incidence of OC is significantly associated with various modifiable risk factors, including smoking, alcohol consumption, and HPV infection.^[17,18]^ These risk factors contribute to the persistent rise of the global burden of OC, presenting a significant challenge to public health systems. Considering the significant impact of OC on socioeconomic and healthcare resources, a comprehensive understanding of its epidemiological trends and the causal relationships of risk factors is essential for formulating effective preventative and intervention strategies.

This study investigated the trends in incidence, mortality, and DALYs of OC globally, regionally, and nationally, utilizing data from GBD spanning from 1990 to 2021. The results indicated that the age-standardized incidence rate and ASRM for OC have consistently increased over the past 30 years, particularly in countries with medium and low-middle quintiles of SDI. In contrast to high-income countries, the prevalence of OC is more severe in low- and middle-income countries. This may pertain to the scarcity of medical services, inadequate early screening, and the widespread occurrence of smoking and unhealthy eating habits in these areas. These findings offer significant evidence for global public health officials, assisting them in modifying health policies based on the burden of OC in their respective countries and regions. Importantly, these descriptions reflect observed epidemiological patterns rather than definitive causal conclusions.

Subsequently, the risk factor analysis of GBD and MR verification suggested that smoking was a significant risk factor for OC. The risk factors of OC are being analyzed for the first time using a combination of MR risk factor analysis and GBD risk factors. The findings align with the current literature. Harmful components in tobacco, including nicotine and tar, have been demonstrated to be carcinogenic. Smoking may facilitate the onset and progression of OC by inducing DNA damage in oral epithelial cells and impairing cellular repair processes. Moreover, smoking can induce persistent inflammatory responses, resulting in the oral mucosa existing in a pro-carcinogenic environment for an extended duration. Studies have demonstrated that the evidence consistent with a likely effect between smoking and OC implies that intervention strategies aimed at modifying smoking behavior should be intensified to decrease the prevalence of OC.^[19,20]^ Policymakers can utilize these data to enhance tobacco control strategies and public health campaigns, particularly in low-SDI countries and regions with a high prevalence of OC. Furthermore, early screening for OC should prioritize high-risk populations, particularly long-term smokers. This will facilitate early lesion detection and intervention, thereby enhancing patient survival rates. However, although MR strengthens causal inference by reducing confounding, its results still rely on key methodological assumptions, and the findings should be interpreted cautiously.

Another innovation of this study lies in that we introduced the autoregressive integrated moving average time series model for the first time to forecast the global burden of smoking-related OC over the next 30 years. The results showed that by 2050, YLDs, DALYs, and the number of death will persistently rise. Nevertheless, these projections should be regarded as scenario-based forecasts rather than precise predictions, as long-term trends may be influenced by future changes in data quality, diagnostic practices, prevention programs, and exposure patterns. This underscores the critical need for enhanced tobacco control measures and serves as a significant reference for policymakers addressing the escalating burden of OC in the future.

Although this study provides a comprehensive analysis of the global burden of OC, certain limitations persist. First, the data quality of the GBD database is contingent upon the reporting accuracy of various countries. Especially in low-income countries, constrained medical resources may result in the underreporting or omission of cases. Second, while the MR analysis may proficiently mitigate the influence of confounding factors, some risk factors did not demonstrate a significant causal relationship in our investigation due to the sample size constraints of some exposure data. In addition, the MR results may still be affected by residual pleiotropy or population structure despite the sensitivity analyses performed. In addition, although smoking has been verified as a major risk factor, other potential risk factors (such as HPV infection and eating habits) have not been fully explored.

Strengthening multidimensional research on OC and its risk factors will not only deepen our understanding of its epidemiological characteristics, but also provide a scientific basis for prevention and control strategies worldwide. With the global prevalence of smoking, the results of this study call for the further strengthening of tobacco control measures in the field of global public health to effectively reduce the incidence and mortality of OC. In the future, continuing to promote research on other potential risk factors will help develop more comprehensive and precise OC prevention strategies.

## 5. Conclusions

The burden of OC remains substantial, especially in low- and middle-income countries, due to limited healthcare services and preventive measures. Strengthening tobacco control, early screening initiatives, and public health campaigns targeting high-risk populations are essential to mitigate the increasing burden of OC. Predictive models indicate an urgent need for comprehensive intervention strategies to curb the future rise of OC worldwide.

## Acknowledgments

We thank all the individuals who contributed to the Global Burden of Disease Study 2021 for their extensive support in finding, cataloguing, and analyzing data and facilitating communication between and among team members.

## Author contributions

**Writing – original draft:** Jiangbo Xin.

**Writing – review & editing:** Weiling Chen, Dongzhen Li, Haoxuan Sun.


